# Anti-TNFα therapy transiently improves high density lipoprotein cholesterol levels and microvascular endothelial function in patients with rheumatoid arthritis: a Pilot Study

**DOI:** 10.1186/1471-2474-13-127

**Published:** 2012-07-23

**Authors:** Aamer Sandoo, Jet J C S Veldhuijzen van Zanten, Tracey E Toms, Douglas Carroll, George D Kitas

**Affiliations:** 1 Department of Rheumatology, Dudley Group of Hospitals NHS Foundation Trust, Russells Hall Hospital, Pensnett Road, Dudley, West Midlands, DY1 2HQ, United Kingdom; 2 School of Sport and Exercise Sciences, University of Birmingham, Birmingham, UK; 3 Arthritis Research UK Epidemiology Unit, University of Manchester, Manchester, UK

**Keywords:** Dyslipidaemia, Anti-TNFα, Endothelial function, Rheumatoid arthritis, Cardiovascular disease

## Abstract

**Background:**

Rheumatoid arthritis (RA) is associated with increased morbidity and mortality from cardiovascular disease (CVD). This can be only partially attributed to traditional CVD risk factors such as dyslipidaemia and their downstream effects on endothelial function. The most common lipid abnormality in RA is reduced levels of high-density lipoprotein (HDL) cholesterol, probably due to active inflammation. In this longitudinal study we hypothesised that anti-tumor necrosis factor-α (anti-TNFα) therapy in patients with active RA improves HDL cholesterol, microvascular and macrovascular endothelial function.

**Methods:**

Twenty-three RA patients starting on anti-TNFα treatment were assessed for HDL cholesterol level, and endothelial-dependent and -independent function of microvessels and macrovessels at baseline, 2-weeks and 3 months of treatment.

**Results:**

Disease activity (CRP, fibrinogen, DAS28) significantly decreased during the follow-up period. There was an increase in HDL cholesterol levels at 2 weeks (*p* < 0.05) which was paralleled by a significant increase in microvascular endothelial-dependent function (*p* < 0.05). However, both parameters returned towards baseline at 12 weeks.

**Conclusion:**

Anti-TNFα therapy in RA patients appears to be accompanied by transient but significant improvements in HDL cholesterol levels, which coexists with an improvement in microvascular endothelial-dependent function.

## Background

Rheumatoid arthritis (RA) is a chronic inflammatory disease, characterised by erosive symmetrical polyarthritis. It is the most common inflammatory arthritis, affecting approximately 0.8% of the adult population. RA is also associated with increased morbidity and mortality from cardiovascular disease (CVD) [1], which appears to be of equal frequency and severity as in those with diabetes mellitus of similar duration [2]. This could be partially attributed to an increased prevalence of traditional CVD risk factors such as hypertension, dyslipidaemia and obesity [3]. In particular, the prevalence of dyslipidaemia in RA is high [4], it is evident in both early [5] and advanced RA [6], and may even associate with some RA susceptibility genes [7] and precede the development of RA [8].

The most common lipid abnormality in RA is reduced levels of high-density lipoprotein (HDL) cholesterol [5,9]. HDL prevents oxidation of low density lipoprotein (LDL) cholesterol and is involved in cellular efflux of accumulated LDL particles in the vasculature, which in turn prevents foam cell accumulation and subsequent endothelial dysfunction (ED) [10]. Inflammation can reduce the anti-atherogenic and anti-oxidant properties of HDL by altering the function of key enzymes important to the metabolism of HDL. For example, pro-inflammatory cytokines such as tumor necrosis factor alpha (TNFα) can reduce hepatic lipase concentration [11]. Hepatic lipase alters the size of HDL particles to allow greater uptake of cholesterol from other cells. Changes to the enzymatic content of HDL (i.e. reduced paraoxonase) also results from inflammation, and transforms HDL into a pro-oxidant and pro-inflammatory molecule [12,13]. Inhibition of TNFα through treatment of RA patients can increase HDL levels [14], as well as reducing disease activity and delaying or even reversing progressive joint damage [15]. Importantly, Anti-TNFα therapy may also reduce cardiovascular mortality in RA [16], possibly as a result of a reduction in inflammation [17].

HDL also exerts specific effects in the vasculature including the stimulation of nitric oxide (NO) in endothelial cells, the reduction of TNFα-mediated superoxide release and repair of damaged blood vessels [18-21]. A number of cross-sectional studies have shown that HDL particle size inversely associates with microvascular and macrovascular ED in a variety of populations including healthy individuals, diabetics [10,22,23] as well as RA patients [24-26]. Furthermore, a systematic review of the literature revealed that HDL levels are inversely associated with the risk for stroke and carotid artery atherosclerosis in various patient groups [27]. Collectively, these findings suggest that HDL may be an important determinant of vascular health in any population, including RA.

The objective of the present longitudinal study was to evaluate the effect of anti-TNFα therapy on HDL cholesterol levels and to explore associations between changes in HDL cholesterol levels with contemporary changes in microvascular and macrovascular endothelial function in RA patients.

## Methods

### Patients

Twenty three consecutive patients with RA who were due to start anti-TNFα therapy on clinical indication (UK guidelines [28]) were recruited from the Rheumatology outpatient clinics of the Dudley Group NHS Foundation Trust, UK. All patients met the retrospective application of the 1987 revised RA criteria of the American College of Rheumatology [29]. The exclusion criterion was: previously confirmed acute coronary syndrome or established CVD. The study had Research Ethics Committee and Research and Development approval from Birmingham, East, North and Solihull Research Ethics Committee and all participants gave their written informed consent according to the Declaration of Helsinki.

### Study protocol

Anti-TNFα patients attended a thermoregulated (22 ± 0.9°C) vascular laboratory after a 12 hour overnight fast between 7:00 am and 11:00 am for 3 separate visits: prior to starting anti-TNFα (pre-treatment), 2 weeks and 3 months after initiation of treatment. Fifteen (65%) of the anti-TNFα patients were started on 40 mg of adalimumab taken fortnightly, six (26%) on 50 mg of etanercept taken weekly and two (9%) on infliximab with a dosage of 3 mg/kg according to standard dose administration protocol. There was no change in any of these medications or their doses during the follow up period. Assessments at 2 weeks and 3 months were performed before the patient received the next dose of their drug. Patients were asked to refrain from exercise 24 hours before the session and from smoking 12 hours before the session.

### Metabolic and serological assessments

All patients underwent evaluation of their medical history and general demographics, examination of height, weight and body mass index (BMI). Blood tests were carried out in the biochemistry laboratories at Russells Hall Hospital, UK. Serum levels of total cholesterol (TC), high-density lipoprotein cholesterol (HDL), triglycerides (TG) and C-reactive protein (CRP) were measured using the Vitros 5.1chemistry system (http://www.orthoclinical.com). Levels of erythrocyte sedimentation rate (ESR) were determined using the Westergren method via a Starrsed Compact (Mechatronics BV, Netherlands). Fibrinogen was measured by photo-optical clot detection based on the Clauss quantitative fibrinogen method, using an automated coagulation analyser (ACL Futura Plus, Instrumentation Laboratory, UK). On each occasion disease activity was assessed with disease activity score in 28 joints (DAS28), along with functional ability using Anglicised version of the Stanford Health Assessment Questionnaire (HAQ), and physical activity levels using International Physical Activity Questionnaire (IPAQ) [30-32]. Concentration of the TNFα cytokine was measured from stored serum samples using FlowCytomix kits (BenderMed Systems GmbH, Vienna, Austria). Fluorescent beads were coated with antigen-specific antibodies for the cytokine and incubated with the sample. The assay follows the same principle as a sandwich immunoassay. Flow-cytometry (Bender MedSystems GMBH, Vienna, Austria) was used to differentiate bead populations by their size and fluorescent signature.

### Microvascular endothelial function

Endothelial function of the microvasculature was assessed non-invasively by a single observer (AS), using Laser Doppler Imaging (LDI) (Moor LDI 2 SIM, Moor Instruments Ltd, Devon, UK) with iontophoresis of 1% acetylcholine (ACh, endothelium-dependent) and 1% sodium-nitroprusside (SNP, endothelium-independent) (Sigma Chemical Co, Montvale, New Jersey, USA) in 0.5 ml of saline. The technique was performed according to previously established guidelines [33] and has been described in detail previously [34]. Briefly, after a baseline scan, ten scans were recorded during iontophoresis of the vasoactive agents using a 30 μA current, followed by two scans during recovery. This technique has an intra-observer co-efficient of variation (CV) for ACh and SNP of 6.5% and 5.9% respectively in our laboratory.

### Macrovascular endothelial function

Assessment of macrovascular endothelial-dependent function was performed using flow-mediated dilatation (FMD) with high-resolution ultrasonography of the brachial artery (Acuson Antares ultrasound system, Siemens PLC, Camberley, UK) according to previously established guidelines [35] and has been described in detail previously [34]. Following ten minutes of rest, endothelium-independent responses were examined by administration of 500 microgram sublingual glyceryl-trinitrate (GTN) tablet (Alpharma, Barnstaple, UK) while the brachial artery was imaged continuously for five minutes. The intra-observer CV was 10.7% for FMD and 11.8% for GTN assessments respectively. For all vascular tests, endothelial function was expressed as the percentage increase in perfusion or diameter from baseline, and all analysis was carried out offline by AS who was blinded to the identity and time point of the patient.

### Statistical analysis

Statistical analysis was performed using SPSS18 (SPSS Inc, Chicago, Illinois). Variables were tested for normality by the Kolmogorov-Smirnov test. Means and standard deviations (SD) were calculated for normally distributed continuous variables and proportions for categorical variables. Log transformation was performed for positively skewed variables as appropriate. The effect of anti-TNFα treatment on blood lipid parameters and vascular indices (continuous variables) was tested using repeated measures analyses of variance (ANOVA). Where appropriate, LSD post hoc analyses were conducted. The change in lipid and inflammatory parameters as well as endothelial function at 2 and 12 weeks was calculated by subtracting the pre-treatment baseline values from the values obtained at 2 and 12 weeks. Pearson correlations were used to examine whether changes in endothelial function related to changes in lipids or disease-related inflammation. Endothelial function and lipid parameters did not differ between the three different types of anti-TNFα treatment at any time point, therefore all treatments were analysed together.

## Results

### Baseline characteristics

The baseline characteristics as well as rheumatologic and cardiovascular medications for study patients are shown in Table 1. There was no change in any of these drugs or their dose during the follow-up period.

**Table 1:** Baseline characteristics of the RA patients.

	**RA Patients**
***General Characteristics***	
Age (years)	54 ± 15
Sex female N (%)	15 (65)
Height (cm)	165 ± 8
BMI (kg/m^2^)	30 ± 6
***Disease-related Characteristics***	
RF Positive N (%)	20 (87)
Disease duration (years)	11 ± 11
***CVD Risk Factors***	
Family history of CVD	12 (40)
Diabetes	1 (3)
Hypertension	6 (20)
High Cholesterol	6 (20)
Insulin Resistance	10 (33)
Overweight	7 (23)
Obese	13 (43)
*Smoking Status*	
Never smoked	11 (48.8)
Previous smokers	7 (30)
Current smokers	5 (22)
***Rheumatologic Medications***	
Methotrexate (%)	16 (70)
Glucocorticosteroids (%)	6 (26)
Medium dose GC (≥7.5 mg) (%)	13 (56)
NSAIDs (%)	6 (26)
***Cardiovascular Medications***	
Anti-hypertensive treatment (%)	5 (22)
Anti-hypercholesterolemic (%)	4 (17)

Results are expressed as Number (%) and mean ± standard deviation. Diabetes = fasting glucose >7 mmol/l and/or oral hypoglycaemic medication or insulin use; hypertension = SBP >140 mmHg, DBP >90 mmHg or use of anti-hypertensive’s; high cholesterol = fasting cholesterol >4.1 mmol/l or use of anti-hypercholesterolemics; insulin resistance = homeostasis model assessment ≥2.5 or quantitative insulin sensitivity check index ≤0.333; overweight: BMI ≥ 23-27.9; obese: BMI ≥ 28. BMI: body mass index, RF: rheumatoid factor.

### Treatment with anti-TNFα

Table 2 shows the lipid profile, disease activity, dyslipidaemia risk factors, and vascular measurements over the course of the study. During follow up of this group, there was, as expected, a significant reduction in all of the markers of disease activity including CRP, ESR, fibrinogen, DAS28 and HAQ. However, ESR levels were no longer significantly different from baseline at 3 months. No changes in TNFα concentration were found in response to treatment. HDL was the only lipid parameter whose levels increased significantly after 2 weeks of treatment, before values returned towards baseline at 3 months (Table 2). The other lipid parameters did not show significant changes in their levels in response to anti-TNFα treatment.

**Table 2:** Lipid profile, disease activity, dyslipidaemia risk factors, and vascular measurements across the three visits for patients starting anti-TNFα therapy.

	**Baseline**	**2 weeks**	**3 months**	**P-value**
**Lipid Profile**				
TC (mmol/l)	4.7 ± 1.0	4.9 ± 1.1	4.8 ± 0.9	0.170
HDL cholesterol (mmol/l)	1.4 ± 0.3	1.5 ± 0.3^†^	1.4 ± 0.3	0.014
TG (mmol/l)	1.4 ± 0.6	1.5 ± 0.7	1.6 ± 0.9	0.236
**Disease Activity**				
CRP (mg/l)	10 (4 to 14)	3 (2.9 to 6)^†^	5 (2.9 to 10)^†^	<0.001
ESR (mmhr)	16 (9–34)	10 (5–21)^†^	17 (5–27)	0.003
Fibrinogen (g/l)	5.1 ± 1.0	4.2 ± .75 ^†^	4.3 ± .91^†^	<0.001
DAS 28	4.22 ± 0.94	2.71 ± 1.38^†^	2.80 ± 1.30^†^	<0.001
HAQ	2.1 ± 0.5	1.3 ± 0.9 ^†^	1.3 ± 0.9 ^†^	<0.001
TNFα cytokine (pg/ml)*	3.27 (0–65)	16.2 (0–73)	33.1 (0–68)	0.452
**Dyslipidaemia Risk Factors**				
BMI (kg/m^2^)	30.2 ± 6.1	30.3 ± 6.1	30.3 ± 6.2	0.747
IPAQ (met min/wk)	2850 ± 5451	2914 ± 3551	2503 ± 3163	0.625
**Vascular Measurements**				
Microvascular endothelial-dependent and endothelial-independent function				
ACh (%)	319 ± 217	437 ± 247 ^†^	348 ± 209	0.017
SNP (%)	252 ± 126	288 ± 150	261 ± 152	0.314
Macrovascular endothelial-dependent and endothelial-independent function				
FMD (%)	9.4 ± 6.9	12.0 ± 9.6	12.0 ± 8.1	0.214
GTN (%)	22.6 ± 7.5	23.2 ± 7.0	23.8 ± 7.2	0.737

Results expressed as mean ± SD or median (interquartile range) as appropriate.

TC: total cholesterol, HDL: high density lipoprotein, TG: triglycerides, CRP: C-reactive protein, ESR: erythrocyte sedimentation rate, DAS28: disease activity score, HAQ: health assessment questionnaire, TNFα: tumor necrosis factor alpha, BMI: body mass index, IPAQ: international physical activity questionnaire, ACh: acetylcholine-mediated dilatation, SNP: sodium nitroprusside-mediated dilatation, FMD: flow-mediated-dilatation, GTN: glyceryl trinitrate-mediated dilatation. ^†^p < 0.05 when compared to baseline visit. *Data for 21 patients only.

Of the vascular measurements, only microvascular endothelial-dependent function (ACh) exhibited a significant change (increased dilatation) after 2 weeks of anti-TNFα therapy compared to baseline measurements: 437 ± 247% vs. 319 ± 217% respectively, *p* = 0.001 (Table 2). None of the other vascular assessments demonstrated significant changes either at 2 weeks or 3 months of follow-up (Table 2). Pearson correlations did not reveal any significant associations between change in microvascular endothelial function and change in HDL cholesterol at 2 weeks (*r* (21) = -.03, *p =* .89). There were no significant correlations between changes in HDL cholesterol with changes in other vascular or inflammatory parameters.

## Discussion

The present study revealed a transient improvement in HDL cholesterol levels following 2 weeks of treatment with anti-TNFα theraphy which returned to baseline levels after 3 months in RA patients with active disease who had newly started anti-TNFα treatment. A concomitant increase in microvascular endothelial-dependent function but not macrovascular endothelial-dependent function was also observed in these patients.

To our knowledge the current study is the first to report the effects of anti-TNFα in RA patients who underwent simultaneous measurements of their lipid profile as well as microvascular and macrovascular endothelial function. Previous studies that measured lipid parameters and endothelial function in response to anti-TNFα treatment have examined endothelial function in the macrocirculation only [36] or have focused on drugs targeting other inflammatory pathways [37,38], and report conflicting findings. Irace and colleagues [36] reported a transient increase in macrovascular endothelial-dependent function immediately after infusion of infliximab at 0, 2 and 6 weeks, however, HDL levels decreased. It was suggested that during inflammation, HDL particles bind with TNFα and buffer the cytokine from the circulation. Inhibition of TNFα with infliximab could reduce TNFα-HDL- complexes and consequently reduce circulatory levels of HDL cholesterol [36]. In contrast, an increase in HDL cholesterol levels was reported in RA patients following short-term (2 weeks and 6 weeks) treatment with infliximab which was correlated with reductions in disease-related inflammation [39]. Similarly, administration of the anti-CD20 agent, rituximab, led to a significant improvement in HDL cholesterol levels and macrovascular endothelial-dependent function after 16 weeks [37] and 24 weeks of treatment [40], both of which are time points associated with maximum efficacy for rituximab in reducing inflammation [41]. Inflammation can reduce HDL levels [42] and its core components, such as the anti-oxidant enzyme paraoxonase [12,13]. It is therefore possible that in the present study, the increase in HDL cholesterol reflected the acute reduction in global inflammation at 2 weeks, with stabilisation of inflammation at 3 months resulting in no further changes in the lipid profile [14].

The transient improvement in microvascular endothelial-dependent function mirrored the increase in HDL cholesterol at 2 weeks with the majority of other risk factors (including physical activity levels) remaining unchanged. HDL cholesterol can exert acute effects in the vasculature, including a reduction in TNFα mediated superoxide release, increased production of endothelial progenitor cells, and stimulation of NO in endothelial cells [18-21] all of which increase endothelial dependent vasodilatation [18]. Given that both HDL cholesterol levels as well as its anti-oxidant ability can increase with administration of anti-TNFα in RA [14,43], it is likely that the improvement in microvascular endothelial-dependent function after 2 weeks of treatment with anti-TNFα was mediated by the increased HDL cholesterol levels. Nevertheless, no association was apparent between baseline HDL cholesterol and endothelial function or changes in HDL cholesterol levels and endothelial function following treatment. The absence of any associations in the present study may be due to the small sample size. To our knowledge, no other studies have reported associations with endothelial function and HDL cholesterol in RA. Further work exploring such associations in a larger sample size is required.

The finding that microvascular function, but not macrovascular function improved following treatment might reflect the heterogeneity of these vascular beds from each other [44]. We have previously shown that microvascular and macrovascular endothelial function are independent from each other in patients with RA [45]. The microvasculature makes up a much larger proportion than macrovessels and may therefore respond earlier to changes in CVD risk factors or other injurious stimuli [46]. Indeed, it has been hypothesised that the inflammatory response as a result of hypercholesterolemia may be initiated by endothelial activation in the microvasculature [46]. Consequently, it is possible that even small changes in blood lipids could have a greater effect on microvascular endothelial-dependent function. There is also some evidence that in other clinical conditions like diabetes, microvascular dysfunction develops independently of macrovascular dysfunction [46], and may even contribute to the development of macrovascular disease [47].

The majority of previous studies examining the effect of anti-TNFα on the lipid profile have included patients treated with infliximab [36,48], and there is little evidence on the effects of other anti-TNFα agents. Different anti-TNFα agents have unique molecular structures, modes of action and half lives and could therefore differentially impact on inflammation and the lipid profile [14]. In the current study only two patients were receiving infliximab infusions, with the majority of patients receiving adalimumab (n = 15) or etanercept (n = 6). Consequently, the effect of each agent on the lipid profile and endothelial function could not be characterised due to the small sample size of the study. Further studies which include a large sample of patients on all types of anti-TNFα agents are required to delineate their individual effects on CVD risk factors and endothelial function.

There is evidence that Inflammation might affect the size of lipid particles, as TNFα can increase the HDL particle size reducing its effectiveness in reverse cholesterol transport [11,49]. This has profound implications on the assessment of blood lipids as the size of the HDL particle may be of equal (or greater) importance to HDL cholesterol levels in the circulation. Furthermore, a number of studies have demonstrated that the protein content of HDL particles is altered during chronic RA disease-related inflammation [50,51]. In particular, there is an increase in acute phase proteins and proteins involved in the complement cascade which results in HDL becoming pro-inflammatory in RA [50]. In addition, RA patients exhibiting pro-inflammatory HDL have greater inflammatory proteins within the HDL molecule when compared to patients exhibiting anti-inflammatory HDL and healthy controls, and pro-inflammatory HDL complexes associate with higher DAS28 and presence of erosive disease [51]. These findings highlight the need for future studies which measure the effects of lipid sub-fractions as well as the protein content of HDL molecules on CVD risk in RA.

Even though the sample size was not large, it had sufficient power to detect differences in HDL and vascular parameters. Post hoc power analyses using GPower3 [52] with significance set at .05 revealed that, given the sample size and effect size, the obtained power was .94 to detect differences in HDL cholesterol, .99 to detect differences in microvascular endothelial function, and .98 to detect differences in macrovascular endothelial function. A limitation of the study was the absence of a control group of RA patients not receiving anti-TNFα. It was decided that it would be unethical to withhold treatment to patients with active disease requiring anti-TNFα, and including patients receiving other disease modifying anti-rheumatic drugs would not be suitable, as relative to the anti-TNFα group, these patients would have much lower levels of baseline inflammation.

## Conclusion

In conclusion, the present study revealed that treatment with anti-TNFα resulted in a transient improvement in HDL cholesterol levels and a concomitant improvement in microvascular but not in macrovascular endothelial function. This was possibly mediated by a favourable effect of HDL cholesterol on microvascular endothelial function. Further prospective studies utilising large sample sizes are required to confirm these findings.

## Abbreviations

ACh: Acetylcholine; Anti-TNFα: Anti-tumor necrosis factor alpha; BMI: Body mass index; CRP: C-reactive protein; CVD: Cardiovascular disease; CV: Co-efficient of variation; DAS28: Disease activity score in 28 joints; ED: Endothelial dysfunction; ESR: Erythtrocyte sedimentation rate; FMD: Flow-mediated dilatation; GTN: Glyceryl-trinitrate mediated dilatation; HAQ: Health Assessment Questionnaire; HDL: High density lipoprotein cholesterol; IPAQ: International physical activity questionnaire; LDI: Laser Doppler imaging; LDL: Low density lipoprotein cholesterol; NO: Nitric oxide; RA: Rheumatoid arthritis; TC: Total cholesterol; TG: Triglycerides; UK: United Kingdom.

## Competing interests

The authors declare that they have no competing interests.

## Authors contributions

AS participated in the design of the study, recruited patients, performed the vascular assessments, conducted data analysis and drafted the manuscript. JVvZ participated in the design of the study, helped with data analysis and in drafting the manuscript. TET helped with data analysis. DC participated in the design of the study, helped with data analysis and in drafting the manuscript. GK participated in the design of the study, helped with data analysis and in drafting the manuscript. All authors have read and approved the final manuscript.

## Pre-publication history

The pre-publication history for this paper can be accessed here:

http://www.biomedcentral.com/1471-2474/13/127/prepub
